# A Kawasaki-like illness in an adult with recent SARS-CoV-2 infection

**DOI:** 10.1093/rap/rkab035

**Published:** 2021-05-18

**Authors:** Deepak Nagra, Mark D Russell, Stefania Rosmini, Daniel Sado, April Buazon, Taimur Shafi, Elizabeth Hamlyn, Gurjinder Sandhu, Andrew I Rutherford, James B Galloway

**Affiliations:** 1 Centre for Rheumatic Disease, King’s College London; 2 King’s College Hospital NHS Foundation Trust, London, UK

Key message

- Kawasaki-like illnesses are rare but important diagnoses to consider in adults with recent coronavirus disease 2019.

Dear Editor, Paediatric multisystem inflammatory syndrome temporally associated with severe acute respiratory syndrome coronavirus 2 (SARS-CoV-2) (PIMS-TS) is an increasingly recognized entity, with features of both Kawasaki disease and toxic shock syndrome. There have been multiple reports of this syndrome in the paediatric population [1, 2]. We describe an adult male presenting with features suggestive of Kawasaki disease after recently confirmed infection with SARS-CoV-2.

A 30-year-old man of Sri Lankan ethnicity presented to our Emergency Department 1 month after PCR-confirmed coronavirus disease 2019 (COVID-19). His initial COVID-19 symptoms had been cough, fever and fatigue, with resolution of these symptoms 2 weeks after onset. Subsequent PCR tests for SARS-CoV-2 were negative.

At presentation to our Emergency Department, the patient reported 7 days of headaches, fever, abdominal pain and vomiting. He had no other localizing symptoms, including no cardiorespiratory symptoms. He was previously fit and well, and he did not take any medications. He had moved to the UK from Sri Lanka in 2015. There was no recent history of travel or known infectious contacts.

At presentation, he had a heart rate of 123 beats/min, respiratory rate of 22 breaths/min, blood pressure of 90/53 mmHg, oxygen saturation of 99% on air, and temperature of 38.0°C. His Glassgow Coma Scale was 15/15. Examination revealed bilateral conjunctival injection (Fig. 1A) and cracking of the lips (Fig. 1B). Full systems examination was otherwise unremarkable, including no evidence of a rash or palpable lymphadenopathy.

**Fig. 1:**
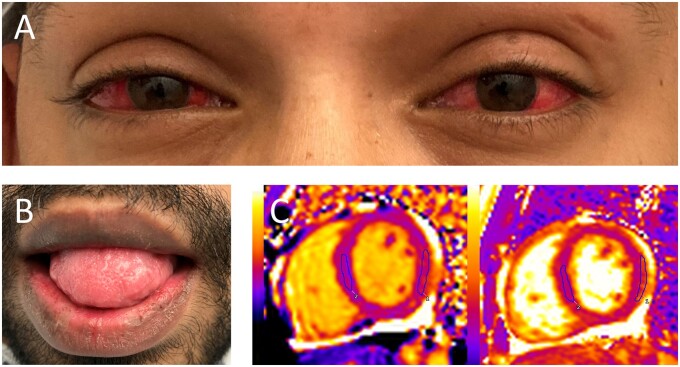
Conjunctival and oral manifestations and cardiac MRI findings in a Kawasaki-like illness after coronavirus disease 2019 (**A**) Bilateral conjunctival injection. (**B**) Cheilitis at presentation. (**C**) Cardiac MRI demonstrating features of active myocardial inflammation, with high native myocardial T1 signal at the basal to mid-inferolateral wall (left image) and matched elevated T2 signal (right image).

Blood tests at presentation showed: white cell count 15.1 × 10^9^/l, neutrophil count 13.5 × 10^9^/l, lymphocyte count 0.96 × 10^9^/l, haemoglobin 154 × 10^9^/l, platelet count 248 × 10^9^/l, CRP 396 mg/l, sodium 128 mmol/l, creatinine 103 μmol/l, aspartate transaminase 48 IU/l and alanine phosphatase 117 IU/l. There were elevations in troponin T (259 ng/l), D-dimer (3620 ng/ml), N-terminal pro B-type natriuretic peptide (NT-pro-BNP) (911 ng/l) and ferritin (2609 ng/l). ANA, anti-dsDNA and ANCA were negative, and there was no evidence of complement consumption. Tests for HAV, HBV, HCV, EBV, CMV, leptospirosis, syphilis, rubella and parvovirus were negative. Blood and urine cultures were sterile. SARS-CoV-2 PCR was negative on admission to the Emergency Department, a chest radiograph was normal, and SARS-CoV-2 IgG was positive. A 12-lead ECG showed sinus rhythm with T-wave inversion in leads III and V3–V5.

Despite broad-spectrum i.v. antibiotics and fluid resuscitation, the patient remained persistently febrile and hypotensive. CT pulmonary angiography showed no pulmonary emboli but demonstrated mediastinal lymphadenopathy and small bilateral pleural effusions. A cardiac MRI demonstrated a small, localized pericardial effusion, preserved left ventricular function, focal hypokinesia at the mid-inferolateral wall and features of active myocardial inflammation (Fig. 1C): high native myocardial T1 signal at the basal to mid-inferolateral wall (1185 ms; normal values, 950–1100 ms using MOLLI sequence at 1.5 T) and matched elevated T2 signal (58 ms; normal values <50 ms) with normal T1 and T2 values in the remote septal myocardium.

A rheumatology opinion was sought; the constellation of persistent fever, bilateral conjunctival injection, mucosal changes and myocarditis were suggestive of a Kawasaki-like illness. IVIG at 2 g/kg was administered over 2 days, in addition to oral aspirin 900 mg daily. The patient responded well, with rapid normalization of his blood pressure, temperature and other symptoms. He was discharged home on aspirin and ramipril for cardiovascular protection, and he continues to do well. Four weeks after discharge from hospital, the patient noted desquamation of the palms of both hands. CT coronary angiography and repeat cardiac MRI are scheduled.

In this report, we describe the successful treatment of an adult with a Kawasaki-like illness temporally associated with SARS-CoV-2 infection. Many of the features in this case mimic those seen in reports of PIMS-TS, including the gastrointestinal symptoms, conjunctival injection, desquamation, myocarditis and cardiogenic shock at presentation [1, 2]. Our case meets the criteria selected by the Centres for Disease Control and Prevention (CDC) in their report of 27 cases of multisystem inflammatory syndromes in adults associated with SARS-CoV-2 infection: age ≥21 years; current or recently confirmed infection with SARS-CoV-2; severe extrapulmonary organ dysfunction in the absence of severe respiratory illness; and laboratory evidence of severe inflammation [3]. Of note, 63% of cases in the CDC’s report were positive for SARS-CoV-2 at the time of presentation, with typical chest imaging features of COVID-19. It is therefore plausible that direct viral injury might have contributed to many of the presentations in the CDC’s report, reflective of the broad inclusion criteria.

In this case, the onset of symptoms 1 month after the initial SARS-CoV-2 infection, coupled with the negative SARS-CoV-2 PCR test on presentation to the Emergency Department, is strongly suggestive of an immunologically mediated process rather than direct viral insult. Recent national consensus guidelines for PIMS-TS separate the presenting phenotype into Kawasaki disease-like presentations and non-specific presentations that do not meet criteria for Kawasaki disease [4], with the recommended first-line treatment being IVIG. Adopting a similar approach for multisystem inflammatory syndrome in adults with recent COVID-19 seems pragmatic. This case highlights the ability of SARS-CoV-2 to trigger unusual immunological adverse events, and the rheumatology community need to be vigilant for these complications.

*Funding*: No specific funding was received from any bodies in the public, commercial or not-for-profit sectors to carry out the work described in this article.

*Disclosure statement*: J.B.G. receives speaker fees from Abbvie, Biovitrum, BMS, Celgene, Chugai, Gilead, Janssen, Lilly, Novartis, Pfizer, Roche, Sanofi, Sobi and UCB. M.D.R. has received honoraria from Pfizer, Lilly and UCB. The other authors have declared no conflicts of interest.

## Data availability statement

Data are available upon reasonable request by any qualified researchers who engage in rigorous, independent scientific research, and will be provided following review and approval of a research proposal and Statistical Analysis Plan (SAP) and execution of a Data Sharing Agreement (DSA). All data relevant to the study are included in the article.
